# Comparison of the efficacy and safety of FP-1201-lyo (intravenously administered recombinant human interferon beta-1a) and placebo in the treatment of patients with moderate or severe acute respiratory distress syndrome: study protocol for a randomized controlled trial

**DOI:** 10.1186/s13063-017-2234-7

**Published:** 2017-11-13

**Authors:** Geoff Bellingan, David Brealey, Jordi Mancebo, Alain Mercat, Nicolò Patroniti, Ville Pettilä, Michael Quintel, Jean-Louis Vincent, Mikael Maksimow, Markku Jalkanen, Ilse Piippo, V. Marco Ranieri

**Affiliations:** 1 Division of Critical Care, University College London Hospitals, NHS Foundation Trust, 235 Euston Road, London, NW1 2BU UK; 2 The NIHR University College London Hospitals Biomedical Research Centre, University College London Hospitals, NHS Foundation Trust, 235 Euston Road, London, NW1 2BU UK; 3 Department of Intensive Care, Hospital de la Santa Creu I Sant Pau, Carrer de Sant Quintí, 89, 08026 Barcelona, Spain; 4 Service de Réanimation, CHU D’Angers, 4 Rue Larrey, 49100 Angers, France; 5 Dipartimento di Emergenza e Urgenza, Azienda Ospedaliera San Gerardo, Via Giambattista Pergolesi 33, 20052 Monza, Italy; 6 Department of Intensive Care, Helsinki University Hospital, Haartmaninkatu 4, Helsinki, 00290 Finland; 7 Anesthesiology and Operative Intensive Care Medicine, Universitätsmedizin Göttingen, Robert-Koch-Straße 40, 37075 Göttingen, Germany; 8 Department of Intensive Care, Erasme Hospital, Université libre de Bruxelles, Route de Lennik 808, 1070 Brussels, Belgium; 9 Faron Pharmaceuticals Oy, Joukahaisenkatu 6, 20520 Turku, Finland; 10 Department of Anesthesia and Critical Care Medicine, Sapienza University of Rome, Policlinico Umberto I Hospital, Viale del Policlinico 155, 00161 Rome, Italy

**Keywords:** Interferon, ARDS, Vascular leakage, CD73

## Abstract

**Background:**

Acute respiratory distress syndrome (ARDS) results in vascular leakage, inflammation and respiratory failure. There are currently no approved pharmacological treatments for ARDS and standard of care involves treatment of the underlying cause, and supportive care. The vascular leakage may be related to reduced concentrations of local adenosine, which is involved in maintaining endothelial barrier function. Interferon (IFN) beta-1a up-regulates the cell surface ecto-5′-nucleotidase cluster of differentiation 73 (CD73), which increases adenosine levels, and IFN beta-1 may, therefore, be a potential treatment for ARDS. In a phase I/II, open-label study in 37 patients with acute lung injury (ALI)/ARDS, recombinant human IFN beta-1a was well tolerated and mortality rates were significantly lower in treated than in control patients.

**Methods/design:**

In this phase III, double-blind, randomized, parallel-group trial, the efficacy and safety of recombinant human IFN beta-1a (FP-1201-lyo) will be compared with placebo in adult patients with ARDS. Patients will be randomly assigned to receive 10 μg FP-1201-lyo or placebo administered intravenously once daily for 6 days and will be monitored for 28 days or until discharged from the intensive care unit. Follow-up visits will then take place at days 90, 180 and 360. The primary endpoint is a composite endpoint including any cause of death at 28 days and days free of mechanical ventilation within 28 days among survivors. Secondary endpoints include: all-cause mortality at 28, 90, 180 and 360 days; organ failure-free days; length of hospital stay; pharmacodynamic assessment including measurement of myxovirus resistance protein A concentrations; and measures of quality of life, respiratory and neurological function at 180 and 360 days. The estimated sample size to demonstrate a reduction in the primary outcome between groups from 30% to 15% is 300 patients, and the study will be conducted in 70–80 centers in nine countries across Europe.

**Discussion:**

There are no effective specific treatments for patients with ARDS and mortality rates remain high. The results from this study will provide evidence regarding the efficacy of a potential new therapeutic agent, FP-1201-lyo, in improving the clinical course and outcome for patients with moderate/severe ARDS.

**Trial registration:**

European Union Clinical Trials Register, no: 2014-005260-15. Registered on 15 July 2017.

**Electronic supplementary material:**

The online version of this article (doi:10.1186/s13063-017-2234-7) contains supplementary material, which is available to authorized users.

## Background

### Acute respiratory distress syndrome (ARDS)

ARDS is a serious clinical disorder which follows a variety of severe lung insults including, among others, pneumonia, aspiration of gastric contents, non-pulmonary sepsis and major trauma. ARDS is a type of acute diffuse lung injury, characterized by acute lung inflammation with injury to the endothelial barriers and alveolar epithelium of the lung, increased pulmonary vascular permeability, and protein-rich pulmonary edema leading to acute respiratory failure. In the Berlin definition of ARDS (Table 1) [1], severity is graded from mild, through moderate, to severe ARDS.

**Table 1:** The Berlin ARDS definition [1]

Characteristic	Mild ARDS	Moderate ARDS	Severe ARDS
Timing	Acute onset within 1 week of a known clinical insult or new or worsening respiratory symptoms		
Hypoxemia	PaO_2_/FiO_2_ > 200– ≤ 300 mmHg with PEEP or CPAP ≥ 5 cmH_2_O	PaO_2_/FiO_2_ > 100– ≤ 200 mmHg with PEEP ≥ 5 cmH_2_O	PaO_2_/FiO_2_ ≤ 100 mmHg with PEEP ≥ 5 cmH_2_O
Origin of edema	Respiratory failure associated with known ARDS risk factors and not fully explained by cardiac failure or fluid overload. An objective assessment of cardiac failure or fluid overload is needed if no ARDS risk factors are present		
Radiological abnormalities (chest X-ray or CT scan)	Bilateral opacities not fully explained by effusions, nodules, masses or lobar/lung collapse		

*ARDS* acute respiratory distress syndrome, *CPAP* continuous positive airway pressure, *CT* computed tomography, *PaO*_*2*_ */FiO*_*2*_ partial pressure of oxygen/fraction of inspired oxygen, *PEEP* positive end-expiratory pressure

Although mortality from ARDS has decreased in the last decade due to improvements in supportive care and in the treatment of underlying conditions [2, 3], it remains high at levels of 20 to 40% across all severities and even higher when associated with dysfunction in other organs [1, 2, 4, 5].

ARDS is also costly in health economics terms. Patients with ARDS consume significantly more resources than other groups of critically ill patients [6] because they have longer intensive care unit (ICU) and hospital stays. The quality of life (QoL) of these patients may also be significantly impacted [7], with 35% of patients with moderate or severe ARDS unable to return to work 24 months after hospital discharge [8, 9].

There are currently no approved pharmacological therapies for ARDS and treatment relies on management of the underlying cause and supportive care.

### Interferon beta-1a

A key pathophysiological feature of ARDS is increased vascular leakage, which has been suggested to be related to a lack of local adenosine which acts to enhance endothelial barrier function [10]. Therefore, any biological substances that can increase local adenosine levels may reduce vascular leakage and thus be of benefit in ARDS. Such a substance is cluster of differentiation 73 (CD73) – a cell surface ecto-5′-nucleotidase enzyme that converts soluble AMP into locally active adenosine [11]. Interferons, such as interferon (IFN) beta-1a, have been shown to up-regulate CD73 and could, therefore, represent a potential treatment for ARDS. Preclinical studies have shown that CD73 expression on endothelial cells is up-regulated by IFN beta-1a treatment in a time- and dose-dependent fashion [12–14]. Furthermore, IFN beta-1a treatment has been shown to prevent leakage in animal models of acute lung injury (ALI) [12]. Enhanced adenosine production also controls leukocyte infiltration [15], thus reducing the escalation of inflammation in the lungs.

Recombinant human IFN beta-1a (FP-1201-lyo) was assessed for the treatment of ALI and ARDS, as defined using the AECC definitions [16], in a phase I/II study [13]. This open-label study, conducted in eight ICUs in the UK, included 37 patients with ALI/ARDS and the optimum tolerated dose of FP-1201-lyo was shown to be 10 μg daily. The primary efficacy endpoint, 28-day mortality, was 8% in the treated patients compared to 32% in the control patients; treatment with FP-1201-lyo was associated with an 81% reduction in the odds of death at 28 days (odds ratio 0.19 (95% CI 0.03–0.72); *p* = 0.01) [13]. The beneficial effects on outcomes were still present at 6-month follow-up. Pyrexia was the most common drug-related treatment-emergent adverse event in the study. All pyrexia events resolved rapidly without sequelae. There were no other safety concerns during the study period. Subcutaneous recombinant human IFN beta-1a is already an approved treatment for patients with relapsing-remitting multiple sclerosis and its safety profile in such patients is well characterized.

Following the promising results of the phase I/II study [13], the current phase III study was designed to confirm the beneficial effects of FP-1201-lyo in a larger population of patients with ARDS. Herein, we describe the final protocol (version 6.0; 29 Aug 2017) for this study, written in accordance with the Standard Protocol Items: Recommendations for Interventional Trials (SPIRIT) guidelines. The SPIRIT Checklist is provided as Additional file 1.

## Methods/design

This is a multicenter, phase III, double-blind, randomized, parallel-group comparison study of the efficacy and safety of FP-1201-lyo compared with placebo in adult patients with moderate or severe ARDS. The study will be conducted in 70–80 centers in nine European countries. The study is registered on the European Union Clinical Trials Register, no: 2014-005260-15. We will conduct the study in accordance with the principles of the Declaration of Helsinki [17] and the International Conference on Harmonization (ICH) guidelines on Good Clinical Practice. The Local Ethics Committee for each center will approve the study (approvals already in place shown in Additional file 2).

### Study population

Study site investigators will screen patients in the ICU for eligibility and obtain written informed consent. Study sites will review all ICU patients daily in order to identify potential patients.

Inclusion criteria include:

1. Patient is aged ≥ 18 years
2. Patient's trachea is intubated and they are receiving mechanical ventilation
3. Patient has a diagnosis of moderate or severe ARDS according to the Berlin definition of ARDS [1]:
  - Acute onset of respiratory failure within 1 week of a known clinical insult or new or worsening respiratory symptoms
  - Respiratory failure associated with known ARDS risk factors and not fully explained by either cardiac failure or fluid overload (an objective assessment of cardiac failure or fluid overload is needed if no risk factors for ARDS are present)
  - Radiological abnormalities on chest X-ray or on computed tomography (CT) scan, i.e., bilateral opacities that are not fully explained by effusions, nodules, masses or lobar/lung collapse
  - Hypoxemia:
    Moderate ARDS: PaO_2_/FiO_2_ > 100 mmHg (>13.3 kPa) to ≤ 200 mmHg (≤ 26.6 kPa) with positive end-expiratory pressure (PEEP) ≥ 5 cmH_2_O
    Severe ARDS: PaO_2_/FiO_2_ ≤ 100 mmHg (≤ 13.3 kPa) with PEEP ≥ 5 cmH_2_O
  - The radiological and hypoxemia criteria (3 (c) and (d)) must occur within the same 24-h period. The time of onset of ARDS is defined as the time when the last of these two ARDS criteria is met
  - Administration of the first dose of study drug must be planned to take place within 48 h of moderate or severe ARDS diagnosis
  - A signed written informed consent form from the patient or the patient’s personal legal representative or a professional legal representative must be available

Exclusion criteria include:

- Woman known to be pregnant, lactating or with a positive (urine or serum test) or indeterminate (serum test) pregnancy test
- Patient simultaneously taking part in another pharmacotherapy protocol
- Patient not expected to survive for 24 h
- Patient has an underlying clinical condition where, in the opinion of the investigator, it would be extremely unlikely that the patient would be able to come off ventilation, e.g., motor neurone disease, Duchenne muscular dystrophy, or rapidly progressive interstitial pulmonary fibrosis
- Patient has severe chronic obstructive pulmonary disease (COPD) requiring long-term home oxygen therapy or mechanical ventilation (non-invasive ventilation or via tracheotomy) except for continuous positive airway pressure (CPAP) or bi-level positive airway pressure (BIPAP) used solely for sleep-disordered breathing
- Patient has congestive heart failure, defined as New York Heart Association class IV
- Patient has acute left ventricular failure
- Patient has liver failure (Child-Pugh grade C)
- Patient has received any prior IFN
- Patient has known hypersensitivity to natural or recombinant IFN beta or to any of the excipients
- Patient is receiving renal dialysis therapy for chronic renal failure
- Patient is receiving extracorporeal membrane oxygenation, high-frequency oscillatory ventilation (HFOV) or any form of extracorporeal lung support
- Patient has had any form of mechanical ventilation (invasive or non-invasive, excluding CPAP alone) for longer than 48 h prior to the diagnosis of ARDS. Non-invasive ventilation has to be continuously applied for at least 12 h per day in these 48 h
- Patient has burns to ≥ 15% of their total body surface area

### Study drug

All study drugs will be supplied in identical vials and will be similar in color and appearance, enabling double-blind conditions. The powdered FP-1201-lyo or placebo (powdered lyophilisate) will be diluted in water for injection near the patient/in the ICU using a MixJect® transfer device (West Pharmaceutical Services GmbH, Germany/Medimop Medical Projects, Ra’anana, Israel). Once prepared, the dose must be administered to the patient immediately. The diluted FP-1201-lyo or placebo will be administered as an intravenous bolus injection via a central or peripheral line. The injection will be followed with a 5-mL flush of sterile saline. FP-1201-lyo and placebo injections will be given once daily for 6 days. The injection will be given at the same time each day ± 1 h providing the patient’s condition allows this. If for any reason this is not possible, the treatment window may be extended by up to 4 h. Subsequent doses should not be delayed and should revert to the original time schedule. No dose modifications or temporary cessations of study drug administration are allowed. If a delay beyond the 4-h window described above is required, the patient must be withdrawn from study drug but all data must continue to be collected per protocol.

### Study protocol

Local study investigators will use an interactive, web-response system to randomize the patients on a 1:1 basis using country and ARDS severity as stratification parameters. To ensure that conclusions are not dominated by data from a small number of centers, and also to obtain a broad spread of patients and centers within the constraints of the inclusion/exclusion criteria, each center will be allowed to include up to, but no more than, 30 patients. Study investigators at each center will enter all study-related data using electronic Case Report Forms (e-CRFs).

**Fig. 1:**
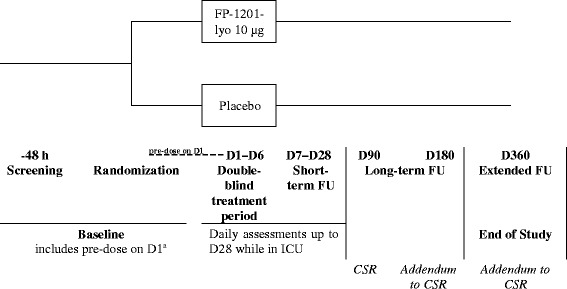
Study design. ^a^Not more than 48 h may elapse between confirmation of moderate or severe acute respiratory distress syndrome (ARDS) during screening and administration of the first dose of study drug on day 1. Once eligibility has been met, randomization can occur during screening or pre-dose on day 1

**Fig. 2:**
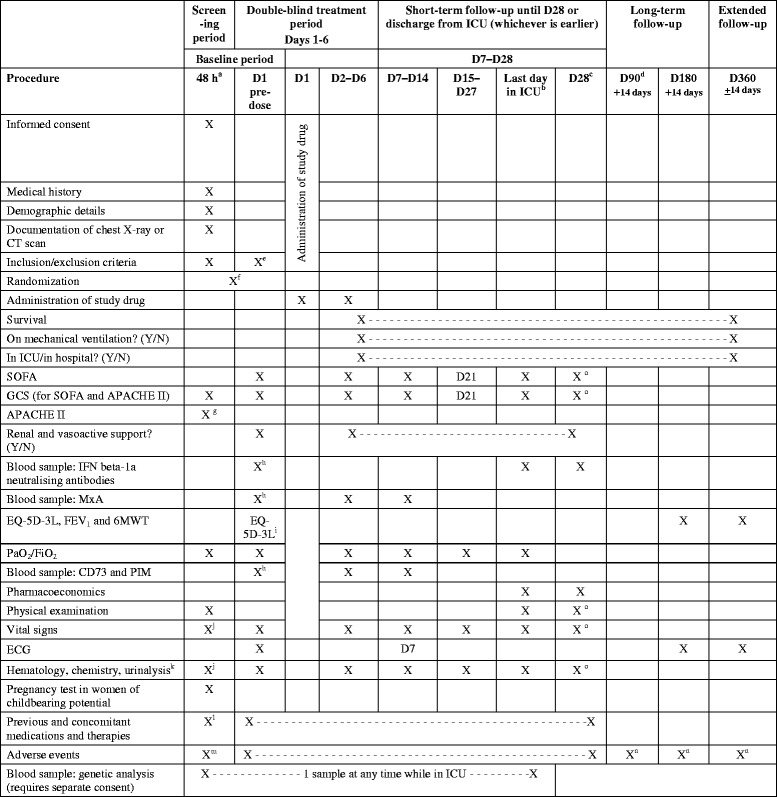
Schedule of procedures. ^a^No more than 48 hours may elapse between confirmation of moderate or severe ARDS and administration of the first dose of study drug. ^b^These assessments will be done on the day the patient leaves the ICU, which will either be on D28 or earlier, according to the clinical progress of the patient. If the patient is still in the ICU on D28, the next visit or telephone contact will be at D90. If a patient leaves the ICU before D28, the survival status and other endpoints must be assessed on D28. ^c^D28 procedures apply for patients leaving the ICU before D28 and for patients withdrawing from the study before D28. For patients withdrawing from the study before D28 a sample should be taken for neutralizing antibodies on the day they leave the ICU. ^d^D90 can either be a visit or telephone contact. ^e^Reconfirm inclusion/exclusion criteria before dosing, including that patient requires mechanical ventilation and is in the ICU. ^f^Randomize after consent obtained and once eligibility criteria confirmed. ^g^Within 24 hours of ICU admission. ^h^1 hour pre-dose. ^i^Baseline EQ-5D-3L to be obtained from relatives and checked later with patient. ^j^For APACHE II scoring. ^k^Samples should be taken in the morning between 04:00 and 10:00. ^l^Medicines and therapies in previous month. ^m^Adverse events will be recorded after informed consent is obtained. ^n^Deaths are reported as SAE. ^0^if it is possible to be performed by the investigator. *APACHE II* Acute Physiology and Chronic Health Evaluation, *CD* Cluster of differentiation, *CT* Computerized tomography, *D* Study day, *ECG* Electrocardiogram, *EQ-5D-3L* EuroQol *5*-Dimensions *3*-Levels questionnaire, *FEV1* Forced expiratory volume in 1 second, *GCS* Glasgow Coma Scale, *ICU* Intensive care unit, *IFN* Interferon, *MxA* Myxovirus resistance protein *A*, *PaO2/FiO2* Partial pressure of oxygen/fraction of inspired oxygen, *PIM* Potential inflammatory marker, *SOFA* Sequential Organ Failure Assessment, *6MWT* 6-minute walk test

Following randomization, patients will receive FP-1201-lyo 10 μg or placebo administered intravenously as a bolus each day for 6 days. The first dose of drug must be administered within 48 h after diagnosis of moderate or severe ARDS. The APACHE II score [18] will be calculated within 24 h of ICU admission. Patients will undergo daily assessments while in the ICU for a maximum of 28 days to include blood sampling for hematology and biochemical variables, CD73 and myxovirus resistance protein A (MxA) concentrations; dipstick urinalysis; PaO_2_/FiO_2_; fluid balance; vital signs (heart rate, respiratory rate, arterial blood pressure, body temperature); and Sequential Organ Failure Assessment (SOFA) score variables [19]. The SOFA score will be calculated pre-dose on day 1, then daily up to day 14, at day 21 and at day 28 while the patient is in the ICU, based on worst daily values. Concomitant medications will be recorded. Blood samples for IFN beta-1a neutralizing antibodies will be taken 1 h prior to the first dose and on the day that the patient leaves the ICU or on day 28 if the patient is still on the ICU. Long-term follow-up will occur at day 90 (visit or telephone contact) and day 180. On day 180 (± 14 days), the EuroQol 5-Dimensions 3-Levels questionnaire (EQ-5D-3 L), the forced expiratory volume in 1 s (FEV_1_) and the 6-minute Walk Test (6MWT) will be assessed. The Clinical Study Report (CSR) will be completed once all the unblinded data up to day 90 have been collected and verified and analyzed according to the statistical plan. Long-term and extended follow-ups will take place at day 180 and day 360 and will include assessments of 6- and 12-month mortality, EQ-5D-3 L, 6MWT and FEV_1_. The extended follow-up data will be reported as addendums to the CSR. During the long-term and extended follow-up periods, and after the end of the extended follow-up visit, patient care will follow normal hospital procedures. The study will be completed when the final patient completes their day 360 study assessment. No interim analyses are planned between days 1–180. The study design is summarized in Fig. 1 and timings of assessments and procedures detailed in Fig. 2.

### General patient management

Apart from administration of the study drug, patients will be managed according to best practice as detailed in Additional file 3. In particular, mechanical ventilation should be provided using a lung-protective ventilation approach incorporating a low-tidal-volume strategy [20]. Any mode of ventilation capable of delivering the prescribed tidal volume (6 mL/kg predicted body weight (PBW) ± 2 mL/kg) within the pressure limitation (plateau pressure limitation ≤ 30 cmH_2_O) may be used. The use of extracorporeal membrane oxygenation (ECMO) is allowed as rescue therapy. Weaning should follow local ICU protocols if available or the guidelines given in Additional file 3. Fluid management should be unrestricted during episodes of shock, but in patients not in shock, a conservative fluid approach should be adopted (see the table in Additional file 3) [21].

### Adverse events

All serious adverse events (SAEs) that occur between the signing of informed consent and day 28 will be recorded. Events occurring after day 28 will be reported only if they are considered to be causally related to the investigational drug; however, all deaths up to day 180 will be reported as SAEs. An SAE is defined as any untoward medical occurrence that at any dose:

- Results in death
- Is life-threatening
- Requires inpatient hospitalization or prolongation of existing hospitalization
- Results in persistent or significant disability or incapacity
- Causes a congenital anomaly or birth defect
- Is an important medical event that may not be immediately life-threatening or result in death or hospitalization but that may jeopardize the patient or require intervention to prevent one of the above outcomes

The study design incorporates an Independent Data Monitoring Committee (IDMC) comprising four members: one independent biostatistician and three senior clinicians with significant experience in ARDS who are not involved in the study. The IDMC will review ongoing safety data in an unblinded manner with meetings scheduled to take place after the data has been received from the last patient of approximately 30, 60, 120, 200 and 300 patients who either have completed 14 days in the study following their first dose of study medication or have been withdrawn for any reason (including death). At each meeting, the IDMC will make a blinded recommendation to the sponsor regarding the study to continue without change, modify study or enrollment to be placed on hold, or study termination.

### Outcome measurements

The primary endpoint is a composite of death and days free of mechanical ventilation within 28 days among survivors. A patient will be considered as ventilator free after two consecutive calendar days of unassisted breathing, defined as breathing spontaneously with a face mask, nasal prong oxygen or room air; T-piece breathing; tracheostomy mask breathing; CPAP ≤ 5 cmH_2_O without pressure support or intermittent mandatory ventilation assistance; or use of CPAP or BIPAP solely for sleep apnea management.

Secondary endpoints can be divided into efficacy, safety and exploratory categories.

#### Efficacy

- All-cause mortality assessed at 28, 90, 180 and 360 days
- Days free of organ failure (assessed using the SOFA score), days free of renal support, days free of vasoactive support, days free of mechanical ventilation, number of intensive care unit (ICU)-free days, assessed at day 28 (or on the last day in the ICU if the patient leaves the ICU before day 28)
- Length of hospital stay
- Immunogenicity of FP-1201-lyo assessed by change in level of neutralizing antibodies to IFN beta-1a between baseline and day 28 (or on the last day in the ICU if the patient leaves the ICU before day 28)
- FEV_1_, neurological functioning (6MWT) and QoL (EQ-5D-3 L) assessed at 180 and 360 days

#### Safety

- Adverse events (assessed by seriousness, intensity and causality) up to day 28, and up to day 360 if the investigator considers that there is a causal relationship with the study drug
- Physical examination, vital signs and laboratory results up to day 28 (or last day in ICU if patient leaves the ICU earlier)

#### Exploratory

- Gas exchange (partial pressure of oxygen/fraction of inspired oxygen (PaO_2_/FiO_2_) ratio) during mechanical ventilation as an indicator of improving lung function on treatment. This categorical endpoint is defined as improvement, no change, or worsening in terms of gas exchange (PaO_2_/FiO_2_ ratio) from baseline to day 28
- Change in the concentration of CD73 and MxA and potential inflammatory markers, including interleukin-6 and interleukin-8, from baseline to day 14
- Genetic testing to identify factors that may be involved in the response or nonresponse of diseases to FP-1201-lyo. In patients who have provided separate consent for this procedure, a 10-mL blood sample for deoxyribonucleic acid (DNA) extraction will be collected at any time during the patient’s ICU stay. All such samples will be destroyed 15 years after completion of the study. If the patient withdraws the consent, the sample will be destroyed immediately

### Data collection and management

The sponsor or sponsor’s designee will conduct a site visit to each study center to verify the qualifications of each investigator, inspect the site facilities and inform the investigator of their responsibilities and the procedures for ensuring adequate and correct documentation.

The investigators will be given access to an online, web-based, electronic data-capture system that is compliant with US Food and Drug Administration Title 21 Code of Federal Regulations Part 11. Access rights to the electronic data-capture system will be carefully controlled and configured according to each individual’s role throughout the study. Computerized data-check programs and manual checks will identify any data discrepancies for resolution. All discrepancies must be resolved online directly by the investigator. Only the investigator will be able to enter and correct data in the e-CRF.

All study findings and documents will be regarded as confidential. Patients will be identified on the e-CRF by their patient number and/or birth date, not by name. Documents that identify the patient must be maintained in confidence by the investigator so that the anonymity of participating patients is ensured.

During the study, the Contract Research Organization (CRO) will make regular site visits to review protocol compliance, conduct source data verification, assess drug accountability and management, assess laboratory procedures and ensure that the study is being conducted according to pertinent regulatory and protocol requirements.

The study blind should only be broken in a medical emergency (where knowledge of the study drug received would affect the treatment of the emergency) or as a regulatory requirement (e.g., for serious adverse events or death).

### Withdrawal of consent

Patients may withdraw from the study at any time and for any reason and such a decision will not affect the ongoing care given to the patient. Data recorded up to the point of withdrawal will be included in the study analyses, unless consent for use of the data has also been withdrawn. If a patient requests termination of the administration of the study drug during the treatment period, then the administration of the study drug will be stopped but the patient will continue in the study and all follow-up assessments will be performed. If a patient withdraws consent during or after the treatment period then no further active study assessments will be performed from that time point. However, permission will be sought to access the patient’s medical records to obtain data relevant to the study (e.g., outcome status).

### Statistical analysis

For 90% power and a two-sided Mann-Whitney *U* test at the significance level of 0.05, a total of 272 patients are required based on the following assumptions:

- Mortality rate of 30% in the control group and 15% in the FP-1201-lyo group at day 28
- 20% of patients survive but with zero ventilator-free days in the control group
- A mean difference (FP-1201-lyo minus control) of 3.0 days in mean ventilator-free days where patients who die are assigned a score of 0

However, assuming that 5% of patients will drop out and a further 4% of the remaining patients will not be evaluable for the efficacy analysis, we plan to randomize 300 patients.

The full analysis set (FAS) will consist of all randomized and treated patients. The per-protocol set (PPS) will consist of patients in the FAS excluding those with major protocol violations. We will perform statistical analyses for the primary and secondary endpoints on both the FAS and PPS.

The safety set will consist of all patients who receive at least one dose of the study drug. The safety and tolerability analyses will be based on this analysis set. A patient who receives the wrong treatment according to the randomization will be analyzed for safety and tolerability in the treatment group corresponding to the treatment received.

The non-parametric analysis of the primary composite endpoint, any cause of death at day 28 and days free of mechanical ventilation within 28 days among survivors (VFDsurv), will use a scoring scheme with patients who do better getting a higher score. We will assign a VFDsurv score of − 1 to all patients who die before 28 days. For those patients who survive to day 28 the VFDsurv score will be equal to the number of VFDs calculated according to the above definition. The statistical method for group comparison of this endpoint will be based on the van Elteren test adjusting for the country, ARDS severity and key baseline characteristics. The statistical methodology for the scoring scheme is as set down in Finkelstein and Schoenfeld [22].

In general, data will not be imputed for the primary efficacy analysis or the safety analysis. For other efficacy analyses, where relevant, imputations will use the last-observation-carried-forward method. All statistical tests will be two-sided and will be performed at the significance level of 0.05.

## Discussion

At present, there is no approved pharmacological treatment for ARDS so there is no possible comparator for studies of potential ARDS therapies. The only currently available treatment for ARDS patients is supportive care. Hence, the current approach to ARDS study design should be to show superiority of the investigated study drug over current standard of care. The study drug will be used in addition to supportive care and, therefore, it is most appropriate to use placebo as a comparator. The pathogenesis of ARDS can be divided into three distinct phases – acute (days 1–6), sub-acute (days 7–14) and chronic (day 15 +). Almost all patients who fail to improve or deteriorate after 1 week of ventilation have evidence of lung fibrosis, so that administering treatment beyond 6 days would add little value to patients included in this study. A 6-day dosing regimen was, therefore, selected as the optimal treatment regimen.

The composite endpoint was chosen as likely to be more sensitive than just 28-day mortality to detect an effect signal. In addition, this study has a double-blind design, so the decision to wean the patient from mechanical ventilation should not be influenced by the treatment group. Owing to the randomization procedure and the double-blind nature of this study, the potential bias of the study results is minimized.

If this study demonstrates improved outcomes in patients receiving FP-1201-lyo, this agent will represent the first specific pharmacological treatment for ARDS and a major advance in the management of these patients.

### Trial status

The first patient was enrolled on 28 December 2015 and the study is ongoing.

## Additional files

Additional file 1: SPIRIT Checklist. (PDF 172 kb)

Additional file 2: List of Ethical Committee approvals already in place. (PDF 403 kb)

Additional file 3: Guidelines for general support. (PDF 286 kb)

Additional file 4: Sample Consent Form. (PDF 133 kb)

## Abbreviations

ALI: Acute lung injury
ARDS: Acute respiratory distress syndrome
CD73: Cell surface ecto-5′-nucleotidase
COPD: Chronic obstructive pulmonary disease
CPAP: Continuous positive airway pressure
CT: Computed tomography
FAS: Full analysis set
HFOV: High-frequency oscillatory ventilation
ICU: Intensive care unit
IDMC: Independent Data Monitoring Committee
IFN: Interferon
MxA: Myxovirus resistance protein A
PAS: Per-protocol set
PEEP: Positive end-expiratory pressure
QoL: Quality of life
SOFA: Sequential Organ Failure Assessment
APACHE II: Acute Physiology and Chronic Health Evaluation
CD: Cluster of differentiation
CT: Computerized tomography
D: Study day
ECG: Electrocardiogram
EQ-5D-3L: EuroQol
5: Dimensions
3: Levels questionnaire
FEV1: Forced expiratory volume in 1 second
GCS: Glasgow Coma Scale
A: PaO2/FiO2=partial pressure of oxygen/fraction of inspired oxygen
PIM: Potential inflammatory marker
