# Supplementary material for: Comparison of the efficacy and safety of FP-1201-lyo (intravenously administered recombinant human interferon beta-1a) and placebo in the treatment of patients with moderate or severe acute respiratory distress syndrome: study protocol for a randomized controlled trial
Source: Trials. 2017 Nov 13;18:536. doi: 10.1186/s13063-017-2234-7 (PMC5683224; doi:10.1186/s13063-017-2234-7)
Supplement: Supplementary file 2 — List of Ethical Committee approvals already in place. (PDF 403 kb) [file 13063_2017_2234_MOESM2_ESM.pdf]

Country	Site Name	Ethics Committee Name	Approval Reference Number	Date EC Approval Received
Belgium	Erasme Hospital	Erasme-ULB (Hospital) Ethics Committee	Erasme Reference -P2015/338, EudraCT No:2014-005260-15	30-nov-15
Belgium	CHU Charleroi<br>Site Hôpital Civil Marie Curie	Erasme-ULB(Hospital) Ethics Committee	Erasme Reference -P2015/338, EudraCT No:2014-005260-15	30-nov-15
Belgium	Centre Hospitalier Universitaire de Liège	Erasme-ULB(Hospital) Ethics Committee	Erasme Reference -P2015/338, EudraCT No:2014-005260-15	30-nov-15
Belgium	UZ Gent, Oost-Vlaanderen	Erasme-ULB(Hospital) Ethics Committee	Erasme Reference -P2015/338, EudraCT No:2014-005260-15	30-nov-15
Belgium	CHU Dinant Godinne UCL Namur	Erasme-ULB(Hospital) Ethics Committee	Erasme Reference -P2015/338, EudraCT No:2014-005260-15	30-nov-15
Belgium	UZ Antwerpen	Erasme-ULB(Hospital) Ethics Committee	Erasme Reference -P2015/338, EudraCT No:2014-005260-15	30-nov-15
Belgium	UZ Brussel	Erasme-ULB(Hospital) Ethics Committee	Erasme Reference -P2015/338, EudraCT No:2014-005260-15	30-nov-15
Finland	Helsinki University Hospital	Hospital District of Helsiniki and UUSIMAA(HUS) Research EC, Dept Surgery	Dnro 255/13/03/02/15	23-sept-15
Finland	Tampere University Hospital	Hospital District of Helsiniki and UUSIMAA(HUS) Research EC, Dept Surgery	HUS/1376/2016	23-sept-15
Finland	Kuopio University Hospital	Hospital District of Helsiniki and UUSIMAA(HUS) Research EC, Dept Surgery	HUS/1376/2016	23-sept-15
Finland	Turku University Central Hospital	Hospital District of Helsiniki and UUSIMAA(HUS) Research EC, Dept Surgery	HUS/1376/2016	28-sept-16
France	CHU D'Angers, Pay-de-la-loire	Ouest II EC -Angers	RCB No.- 2014-005266-15,EC Identification No 2015/24	26-nov-15
France	Hôpital Charles-Nicolle, Seine-Maritime	Ouest II EC -Angers	RCB No.- 2014-005266-15,EC Identification No 2015/24	26-nov-15

France	Centre Hospitalier Régional d'Orléans, Orléans cedex 2	Ouest II EC -Angers	RCB No.- 2014-005266-15,EC Identification No 2015/24	26-nov-15
France	Chu De Poitiers, Poitou-Charentes	Ouest II EC -Angers	RCB No.- 2014-005266-15,EC Identification No 2015/24	26-nov-15
France	Hôpital Cochin, Réanimation Médicale Hospitalisation, Île-de-France	Ouest II EC -Angers	RCB No.- 2014-005266-15,EC Identification No 2015/24	26-nov-15
France	Pitié-Salpêtrière Hospital, Paris	Ouest II EC -Angers	RCB No.- 2014-005266-15,EC Identification No 2015/24	26-nov-15
France	Hôpital de la Croix Rousse, Rhône	Ouest II EC -Angers	RCB No.- 2014-005266-15,EC Identification No 2015/24	26-nov-15
France	Nouvel Hôpital Civil, Alsace	Ouest II EC -Angers	RCB No.- 2014-005266-15,EC Identification No 2015/24	26-nov-15
France	Centre Hospitalier Universitaire de Bicêtre, Ile-de- France	Ouest II EC -Angers	RCB No.- 2014-005266-15,EC Identification No 2015/24	26-nov-15
France	Hopital Nord de Marseille	Ouest II EC -Angers	RCB No.- 2014-005266-15,EC Identification No 2015/24	21-juin-16
France	CHU de la Cavale Blanche, Bretagne	Ouest II EC -Angers	RCB No.- 2014-005266-15,EC Identification No 2015/24	21-juin-16
France	CHU Bretonneau, Centre Val de Loire	Ouest II EC -Angers	RCB No.- 2014-005266-15,EC Identification No 2015/24	21-juin-16
Germany	Universitätsmedizin Göttingen<br>Klinik für Anästhesiologie,Göttingen	EC UMG, University Medical Centre Gottingen	EC Ref Number: 33/8/15	5-févr-16
Germany	Klinikum Augsburg<br>Klinik für Anästhesiologie,Bayern	EC UMG, University Medical Centre Gottingen	EC Ref Number: 33/8/15	5-févr-16
Germany	Kliniken der Stadt Köln<br>Klinikum Merheim Lungenklinik,Nordrhein-Westfalen	EC UMG, University Medical Centre Gottingen	EC Ref Number: 33/8/15	5-févr-16
Germany	University Medical Center Hamburg-Eppendorf, Hamburg	EC UMG, University Medical Centre Gottingen	EC Ref Number: 33/8/15	5-févr-16

Germany	Universitätsklinikum Leipzig, Sachsen	EC UMG, University Medical Centre Gottingen	EC Ref Number: 33/8/15	5-févr-16
Germany	Universitätsklinikum Bonn Klinik und Poliklinik für Anästhesiologie, Nordrhein-Westfalen	EC UMG, University Medical Centre Gottingen	EC Ref Number: 33/8/15	5-févr-16
Germany	Universitätsklinikum Carl Gustav Carus Dresden, Sachsen	EC UMG, University Medical Centre Gottingen	EC Ref Number: 33/8/15	5-févr-16
Germany	Charite-Universitatsmedizin Berlin	EC UMG, University Medical Centre Gottingen	EC Ref Number: 33/8/15	9-août-16
Italy	Giacomo Bellani, Università degli Studi Milano Bicocca, Dipartimento di Medicina e Chirurgia<br>A.O. San Gerardo, Dipartimento di Emergenza e Urgenza<br>Via Pergolesi 33, Monza (MB), Italy	Ethics Committee of the Province of Monza Brianza	EC Ref 2147	14-oct-15
Italy	Fondazione Policlinico Universitario Agostino Gemelli	Ethics Committee Fondazione Pol. Univ A Gemelli	EC Ref no 2901/16	7-avr-16
Italy	Azienda Ospedaliera Universitaria Maggiore della Carità	Ethics Committee Clinic Corso Novara	EC Ref no 164/15	7-avr-16
Italy	AOU Città della Salute e della Scienza di Torino	Ethics Committee of Torino	EC Ref no:2147	11-juil-16
Italy	Universita degli Studi di Roma “La Sapienza” Roma	Ethics Committee Pol Umberto	EC Ref no 69/16	29-mars-16

Italy	Unità Operativa Rianimazione Generale "Emma Vecla", Dipartimento di Anestesia, Rianimazione ed Emergenza Urgenza, IRCCS Ospedale Maggiore Policlinico, Via Francesco Sforza 28, 20122 Milano	Ethics Committee Milano Area B	EC Ref 176_2016	12-avr-16
Italy	Azienda Ospedaliera Universitaria Sant'Anna , Ferrara	Ethics Committee Della Province Di Ferrara	EC Ref 160899 ,	13-oct-16
Spain	Hospital de la Santa Creu I Sant Pau,Barcelona	EC Fundacio de Gestio Sanitaria, Hosp Santa Creu	EC Ref 15/235 (r)	10-févr-16
Spain	Corporació Sanitària Parc Taulí,Barcelona	EC Fundacio de Gestio Sanitaria, Hosp Santa Creu	EC Ref 15/235 (r)	10-févr-16
Spain	Hospital Clínic i Provincial de Barcelona	EC Fundacio de Gestio Sanitaria, Hosp Santa Creu	EC Ref 15/235 (r)	10-févr-16
Spain	Hospital Universitario Rio Hortega,Valladolid	EC Fundacio de Gestio Sanitaria, Hosp Santa Creu	EC Ref 15/235 (r)	10-févr-16
Spain	Hospital Universitari Vall d'Hebron,Barcelona	EC Fundacio de Gestio Sanitaria, Hosp Santa Creu	EC Ref 15/235 (r)	10-févr-16
Spain	Hospital Universitari Son Espases,Baleares	EC Fundacio de Gestio Sanitaria, Hosp Santa Creu	EC Ref 15/235 (r)	10-févr-16
Spain	Hospital Universitario del Henares,Comunidad de Madrid	EC Fundacio de Gestio Sanitaria, Hosp Santa Creu	EC Ref 15/235 (r)	10-févr-16
Spain	Hospital Universitario de Getafe	EC Fundacio de Gestio Sanitaria, Hosp Santa Creu	EC Ref 15/235 (r)	10-févr-16
Spain	Hospital Universitari Mútua de Terrassa	EC Fundacio de Gestio Sanitaria, Hosp Santa Creu	EC Ref 15/235 (r)	10-févr-16
Spain	Hospital Universitario de Gran Canaria Dr Negrin	EC Fundacio de Gestio Sanitaria, Hosp Santa Creu	EC Ref 15/235 (r)	10-févr-16

United Kingdom	University College London Hospitals, NHS Foundation Trust	London - Hamstead Research Ethic Committee	EC Ref 15/LO/2098, IRAS ID 182147	23-déc-15
United Kingdom	Guy's and St Thomas' NHS Foundation Trust, Adult Intensive Care Unit, First floor, East Wing	London - Hamstead Research Ethic Committee	EC Ref 15/LO/2098, IRAS ID 182147	23-déc-15
United Kingdom	St George's Hospital GICU 1st Floor St James Wing	London - Hamstead Research Ethic Committee	EC Ref 15/LO/2098, IRAS ID 182147	23-déc-15
United Kingdom	Southampton General Hospital / University Hospital Southampton NHS Foundation Trust	London - Hamstead Research Ethic Committee	EC Ref 15/LO/2098, IRAS ID 182147	23-déc-15
United Kingdom	Critical Care Research Office, Golden Jubilee Wing, King's College Hospital / King's College Hospital NHS Foundation Trust	London - Hamstead Research Ethic Committee	EC Ref 15/LO/2098, IRAS ID 182147	23-déc-15
United Kingdom	Queen Alexandra Hospital / Portsmouth Hospitals NHS Trust	London - Hamstead Research Ethic Committee	EC Ref 15/LO/2098, IRAS ID 182147	23-déc-15
United Kingdom	Bristol Royal Infirmary/University Hospitals Bristol Foundation Trust	London - Hamstead Research Ethic Committee	EC Ref 15/LO/2098, IRAS ID 182147	23-déc-15
United Kingdom	St Mary's Hospital, Imperial College Healthcare NHS Trust,	London - Hamstead Research Ethic Committee	EC Ref 15/LO/2098, IRAS ID 182147	23-déc-15
United Kingdom	Charing Cross Hopital/Imperial College Healthcare<br>Fulham Palace Road<br>London<br>W6 8RF	London - Hamstead Research Ethic Committee	EC Ref 15/LO/2098, IRAS ID 182147	23-déc-15
United Kingdom	Charing Cross Hospital/Imperial College Healthcare NHS Trust	London - Hamstead Research Ethic Committee	EC Ref 15/LO/2098, IRAS ID 182147	23-déc-15
United Kingdom	Royal Infirmary of Edinburgh, ICU Research Office, Chancellor Building	London - Hamstead Research Ethic Committee	EC Ref 15/LO/2098, IRAS ID 182147	23-déc-15

United Kingdom	Nottingham University Hospitals NHS Trust, DREEAM, Emergency Department, West Block, A Floor	Health Research Authority	EC Ref 15/LO/2098, IRAS ID 182147	05 Oct 2016
United Kingdom	Norfolk & Norwich Foundation Trust University Hospital	Health Research Authority	EC Ref 15/LO/2098, IRAS ID 182147	12 Dec 2016
United Kingdom	Lancashire Teaching Hospitals NHS Foundation Trust	Health Research Authority	EC Ref 15/LO/2098, IRAS ID 182147	21 Dec 2016
