# Supplementary material for: Comparison of the efficacy and safety of FP-1201-lyo (intravenously administered recombinant human interferon beta-1a) and placebo in the treatment of patients with moderate or severe acute respiratory distress syndrome: study protocol for a randomized controlled trial
Source: Trials. 2017 Nov 13;18:536. doi: 10.1186/s13063-017-2234-7 (PMC5683224; doi:10.1186/s13063-017-2234-7)
Supplement: Supplementary file 3 — Guidelines for general support. (PDF 286 kb) [file 13063_2017_2234_MOESM3_ESM.pdf]

Additional File 3

Guidelines for general support

Ventilator management

Investigative sites must use a lung-protective ventilation approach incorporating a low tidal volume (Vt) strategy according to the ARDS Network publication [1]. Any mode of ventilation capable of delivering the prescribed Vt (6 mL/kg predicted body weight [PBW] ±2 mL/kg) within the pressure limitation (plateau pressure limitation ≤30 cm of water) may be used. If Pplat is ≥30 cmH_2O, then Vt should be reduced to 5 mL/kg PBW and then to 4 mL/kg PBW if necessary to decrease Pplat to ≤30 cmH_2O. The oxygenation target is arterial partial pressure of oxygen (PaO_2) of 7.33–10.67 kPa or peripheral capillary oxygen saturation (SpO_2) of 88–95%. The minimum PEEP that should be used is 5 cmH_2O. It is recommended to raise the ventilator respiratory rate incrementally up to 35 breaths per minute (maximum set rate) if a pH <7.30 is observed. There are no specific rules concerning the ratio of the duration of inspiration to the duration of expiration (inspiration: expiration ratio) settings. The use of bicarbonate is allowed if a pH ≤7.30 is observed. The use of extracorporeal membrane oxygenation is allowed as rescue therapy.

Where there is an ICU weaning policy on ventilation this should be followed. In the absence of a unit policy, the guidelines below should be followed:

- Patients should be assessed for the following weaning readiness criteria each day between 06:00 and 12:00 except during the first 24 hours after randomization.
    a) PaO_2/FiO_2 >300 after 1 hour with PEEP <10 and FiO_2 <0.5
    b) Values of both PEEP and FiO_2 ≤ values from previous day
    c) The patient is not receiving neuromuscular blocking agents
    d) The patient is exhibiting inspiratory efforts
    e) Systolic arterial pressure ≥90 mmHg without vasopressor support (≤5 µg/kg/min dopamine or dobutamine will not be considered vasopressor support in this context)
- If criteria are met, application of respiratory management will be stopped, extra-corporeal carbon dioxide removal will be interrupted, and vascular catheters removed. Clinicians will then be free to use any ventilation protocol they consider clinically appropriate.
- A spontaneous breathing trial (SBT) procedure and assessment for unassisted breathing will be performed. The SBT will take place over up to 120 minutes of spontaneous breathing with FiO_2 <0.5 using any of the following approaches:
    a) Pressure support (PS) <5 cmH_2O, PEEP <5 cmH_2O

b) Continuous positive airway pressure (CPAP) <5 cmH_2O
c) T-piece
d) Tracheotomy mask

- Criteria for reporting a SBT as "successful" are:
    a) $SpO_2 \geq 90\%$ and/or $PaO_2 \geq 60$ mmHg
    b) Respiratory rate ≤35/min
    c) pH ≥7.30
    d) No respiratory distress (defined as ≥2 of the following):
    - Heart rate ≥120% of the rate at 06:00 (≤5 min at ≥120% may be tolerated)
    - Marked use of accessory muscles
    - Abdominal paradox
    - Diaphoresis
    - Marked subjective dyspnoea

If any of criteria a–d are not met, the SBT will be reported as "unsuccessful" and previous ventilator settings will be initiated or PS ≥10 cmH_2O with PEEP and FiO_2 = previous settings. The patient should be reassessed for weaning the following day.

If all criteria a–d are met for the last 30 minutes of the trial, the SBT will be reported as "successful" and ventilation support will be removed.

- Patients will be reported as "ventilator free" after two consecutive calendar days of "unassisted breathing". "Unassisted breathing" will be defined as any of the following:
    a) Spontaneously breathing with face mask, nasal prong oxygen or room air
    b) T-piece breathing
    c) Tracheostomy mask breathing
    d) CPAP ≤5 without PS or intermittent mandatory ventilation assistance
    e) Use of CPAP or bi-level positive airway pressure solely for sleep apnoea management

Fluid management

Fluid management will be unrestricted during episodes of shock. However, in patients not in shock, a conservative fluid approach should be adopted. If the ICU has a fluid balance policy for ventilated patients, this should be followed. If no policy exists, those patients not in shock should be managed with a conservative fluid management approach represented by a simplification of the algorithm used in the ARDS Network FACTT study [2] (Table). If not already being used, this conservative fluid management approach should be initiated within 4

hours of study drug treatment being initiated and should be continued until the patient achieves unassisted breathing or day 7, whichever occurs first.

Table. Conservative Fluid Management Algorithm

<table>
<tr><th rowspan="2">CVP (recommended), cmH_2O</th><th rowspan="2">PAOP (optional), cmH_2O</th><th colspan="2">MAP ≥60 mmHg AND
off vasopressors for ≥12 hours</th></tr>
<tr><th>Average urine output <0.5 mL/kg/h (PBW)</th><th>Average urine output ≥0.5 mL/kg/h (PBW)</th></tr>
<tr><td>>8</td><td>>12</td><td>Furosemide[a]

Reassess in 1 hour</td><td rowspan="2">Furosemide[a]

Reassess in 4 hours</td></tr>
<tr><td>4–8</td><td>8–12</td><td rowspan="2">Give fluid bolus as fast as possible[b]

Reassess in 1 hour</td></tr>
<tr><td><4</td><td><8</td><td>No intervention
Reassess in 4 hours</td></tr>
</table>

[a] Recommended furosemide dosing to begin with 20 mg bolus or 3 mg/h infusion or last known effective dose. Double each subsequent dose until goal achieved (oliguria reversal or intravascular pressure target) or maximum infusion rate of 24 mg/h or 160 mg bolus reached. Do not exceed 620 mg/d. Also, if patient has heart failure, consider treatment with dobutamine.

[b] Recommended fluid bolus= 15 mL/kg (PBW) crystalloid (round to nearest 250 mL) or 1 unit packed red cells or 25 g albumin.

Abbreviations: CVP=central venous pressure; MAP=mean arterial pressure; PAOP=pulmonary artery occlusion pressure; PBW=predicted body weight.

Body Positioning

The use of prone positioning is recommended in severe ARDS (PaO_2/FiO_2 <150 mmHg) as per the PROSEVA study [3].

Nutritional Support

Nutritional support should be initiated as early as possible (total parenteral nutrition or enteral nutrition).

Nitric Oxide

The use of nitric oxide is not recommended.

Corticosteroids

The use of corticosteroids to treat ARDS or sepsis should be avoided.

Glucose Management

Maintaining blood glucose levels in a reasonable range is important. Each investigational site will use its own ICU protocol for glucose and insulin administration. In the absence of a locally agreed ICU best practice protocol, the target should be to maintain blood glucose at 8–10 mMol/L (144–180 mg/dL) as per the control limb of the NICE-SUGAR study [4].

Neuromuscular Blockers

In line with the results of the ACURASYS study [5], neuromuscular blockers can be given to patients with ARDS when their PaO_2/FiO_2 ratio is <120 with a PEEP ≥5 cmH_2O. When given, neuromuscular blockers should be administered for a maximum of 48 hours.

Sedation

The minimum sedation possible will be given; that is, no more sedation than the patient needs. Daily interruption of sedation to permit a detailed assessment of the patient should be considered as part of a weaning protocol, dependent on the individual patient's condition.

Prevention of pneumonia

Efforts to prevent ventilator-associated pneumonia, bacterial or pneumonia cause unspecified, and new nosocomial lung infections should be made by:

a) Administration of a chlorhexidine oral rinse and chlorhexidine gel paste, as this can reduce the oral bacterial load. Several studies have shown that oral decontamination is an effective method of reducing ventilator-associated pneumonia
b) Raising the head of the patient's bed: elevation of the head of the bed to 30° is supported as a preventive strategy that lowers the risk of aspiration

Deep vein thrombosis: thrombosis prevention

Investigators should follow their local guidance for prevention of deep vein thrombosis.

Stress ulcer prevention

Investigators should follow their local guidance for prevention of stress ulcers.

References

1. The Acute Respiratory Distress Syndrome Network. Ventilation with lower tidal volumes as compared with traditional tidal volumes for acute lung injury and the acute respiratory distress syndrome. N Engl J Med. 2000;342:1301-8.

2. Wiedemann HP, Wheeler AP, Bernard GR, Thompson BT, Hayden D, deBoisblanc B et al. Comparison of two fluid-management strategies in acute lung injury. N Engl J Med. 2006;354:2564-75.

3. Guerin C, Reignier J, Richard JC, Beuret P, Gacouin A, Boulain T et al. Prone positioning in severe acute respiratory distress syndrome. N Engl J Med. 2013;368:2159-68.

4. Finfer S, Chittock DR, Su SY, Blair D, Foster D, Dhingra V et al. Intensive versus conventional glucose control in critically ill patients. N Engl J Med. 2009;360:1283-97.

5. Papazian L, Forel JM, Gacouin A, Penot-Ragon C, Perrin G, Loundou A et al. Neuromuscular blockers in early acute respiratory distress syndrome. N Engl J Med. 2010;363:1170-16.
