# Supplementary material for: Comparison of the efficacy and safety of FP-1201-lyo (intravenously administered recombinant human interferon beta-1a) and placebo in the treatment of patients with moderate or severe acute respiratory distress syndrome: study protocol for a randomized controlled trial
Source: Trials. 2017 Nov 13;18:536. doi: 10.1186/s13063-017-2234-7 (PMC5683224; doi:10.1186/s13063-017-2234-7)
Supplement: Supplementary file 4 — Sample Consent Form. (PDF 133 kb) [file 13063_2017_2234_MOESM4_ESM.pdf]

INFORMED CONSENT FORM

INFORMATION TO PATIENT

Short Title: **The INTEREST Study: A clinical trial to investigate interferon beta in the treatments of ARDS**

Title of Study: **A Phase III Double-blind, Randomised, Parallel Group Comparison of the Efficacy and Safety of FP-1201-lyo (Recombinant Human Interferon beta-1a) and Placebo in the Treatment of Patients with Moderate or Severe Acute Respiratory Distress Syndrome**

Protocol Number: **FPCLI002**

Sponsor: **Faron Pharmaceuticals Ltd**
Joukahaisenkatu 6
FIN-20520 Turku
Finland

[ETHICS COMMITTEE (EC) or
INVESTIGATIONAL REVIEW BOARD (IRB):]

[EC/IRB Name]
[EC/IRB Address]
[Office Hours Tel]
[Out of Hours Tel]

Investigator:

[Investigator Name]
[Site Address]
[Office Hours Tel]
[Out of Hours Tel]

Patient Number:

For the Patient: When reading this form, please note that the words “you” and “your” refer to you, the person invited to participate in the study.

For the Legal Representative: This research study may involve patients who do not have the legal capacity or ability to consent to their participation, for example because they are unconscious. If you are a legal representative giving consent on behalf of the patient, “you” and “your” should be read as referring to the person you are legally authorised to represent.

Why are you receiving this information?

You are being invited to take part in a research study. This Patient Information Sheet/Informed Consent Form provides information about the study to help you decide if you would like to participate. It is important that you understand why the research is being done and what it will involve, and the possible benefits, risks and discomforts.

Before you decide, please take time to read the following information carefully and discuss it with others if you wish. If there is anything you do not understand or if you would like more information, please ask the study doctor or study staff.

This clinical research study is being sponsored by Faron Pharmaceuticals Ltd. An independent ethics committee has reviewed the study's safety and ethics to ensure that patients' rights are not violated.

No study-specific procedures will be done until you have read and signed this form.

What is the purpose of this clinical research study?

You are being asked to take part in this study as you have developed a serious lung problem known as the Acute Respiratory Distress Syndrome (ARDS). ARDS develops in patients who are often unwell for other reasons such as pneumonia, severe infections or following a serious injury.

We are investigating a new treatment for ARDS. ARDS is a life threatening condition that affects your lungs, resulting in a dangerously low level of oxygen in your blood. Currently there are no treatments available for this condition apart from using a breathing machine (ventilator) to support you during this time.

We are investigating a new drug treatment for ARDS. “Investigational new drug” means a drug that has not been approved as a marketed product (i.e., available to be prescribed or sold) by any regulatory authorities. It is a recombinant interferon beta-1a (FP-1201-lyo) that may help improve how your lungs work. We hope this would reduce the time you need to be on the ventilator and improve your chances of survival. Currently there are no approved medicines for treating moderate or severe ARDS and your doctors are using a breathing machine (ventilator) to support you during this time. FP-1201-lyo might also reduce the secondary complications (e.g., kidney failure) that occur when the amount of oxygen in the body is too low.

Interferon beta-1a is already an approved treatment for subjects with multiple sclerosis (MS) and any side effects in these subjects are well known. An earlier small study suggested FP-1201-lyo is both safe and effective in patients with ARDS but we need to do this larger study to demonstrate this conclusively. The dose of FP-1201-lyo in this study has been chosen to minimise side effects.

There is an option for you to provide a blood sample for genetic testing. There is a separate information and consent form for this.

About 300 subjects will be taking part in this study at 70 to 80 hospitals around Europe.

Do I have to take part?

Your participation in this study is voluntary. You do not have to take part, and you may discontinue your involvement at any time without penalty or loss of benefits to which you are otherwise entitled. If you decide to leave the study before the last study visit, tell the study doctor and follow instructions. It may be helpful if you could explain your reasons. You may receive standard treatment and no prejudice will be shown towards you for medical care or participation in future research.

In addition, your study doctor or the sponsor (Faron Pharmaceuticals Ltd.) may withdraw you from the study for your own safety, even if you wish to continue to participate, for example:

- the study doctor feels it is in your best interest to discontinue
- you do not follow the study doctor's instructions
- you have a side effect that requires other treatment
- Faron Pharmaceuticals Ltd, the Independent Ethics Committee or regulatory authority stops the study for any reason
- The administration of the treatment has been delayed for any reason beyond 4 hours.

If your participation in the study is stopped early, you may be asked to complete end of study procedures (such as a final medical examination and laboratory tests) for your own safety.

What procedures are involved?

You will be assessed for suitability into the study when you are in the intensive care unit (ICU). Your study doctor will check to see if there are any reasons why you should not take part in the study.

The maximum length of time you are in the study will be a total of 376 days. You will be monitored daily until you are discharged from ICU or to Day 28, whichever is soonest. You are then invited to return for assessments at 3, 6 and 12-month checkpoints.

Study Medication

You will be given a 6-day course of study medication. The medication either FP-1201-lyo or placebo (an injection containing only inactive ingredients) will be given using an existing drip or as an injection into a vein. You will also be given standard care and treatment for ARDS.

This study is “double blinded”. This means that neither you nor the study doctor will know which treatment you are receiving. If necessary, the study doctor can find out which treatment you are receiving.

Which treatment you will be given will be decided randomly, like tossing a coin. About 150 subjects will be given FP-1201-lyo and about 150 will be given placebo. You should not take part in this study if you are not willing to receive placebo.

Study Procedures

Screening.

To find out if you qualify to be in this study, your medical chart will be reviewed to learn about your medical history, what medications you have recently taken, and you will be given a full physical examination. Demographic data such as age, ethnicity and gender are also recorded as well as information on your medical history, and clinical data collected about your participation in the study. Blood gas analysis are used as an indicator of lung function. A chest X-ray or computerised tomography (CT) scan will be performed (if not already done). A blood sample will be taken from an artery to check how you are responding to the treatment and if possible, a urine sample will be collected for laboratory tests. If you are a woman who may be able to have children, these tests will also include a urine or blood pregnancy test. A score to measure the severity of your state of health (called APACHE II score) will be calculated using the results of your physical examination, your vital signs and the results of your blood test all carried out under your standard of care in intensive care. There might be procedures which have been done in the ICU before you sign this form which are normally done in subjects with ARDS. Some of the results from these procedures might be used to find out if you qualify to be in this study, and they will not be repeated after you have signed the form.

Day 1

Day 1 is the first day of dosing. Before you receive the first dose of study medication the study doctor will make sure you are still eligible to take part in the study. You will have a check on the status of your vital organs (brain, heart, lungs, liver, kidneys and blood clotting), and a note will be made of any treatment you are receiving. Your relatives will be asked about your quality of life before you were admitted to the ICU. Your vital signs (respiratory rate, temperature, blood pressure, pulse) will be measured and your medical chart will be reviewed to see if you have had any new illness or injury. In order to check your heart activity, a heart test, called a 12-lead electrocardiogram (ECG) will be performed. Blood gas analysis will be used as an indicator of lung function. A blood sample will be taken to check your general health (the sample will be analysed in the hospital’s laboratory) and to give a baseline check on the effects of the study medication (this sample will be tested for biomarkers in a separate, centralised laboratory), and, if possible, a urine sample will also be collected for laboratory tests.

Once the tests are completed, you will be given the first dose of study medication.

Days 2 to 6

On Days 2 to 6 you will be dosed with study medication (or placebo) once a day.

Days 2 to 28 (or to last day in ICU).

On Days 2 to 28 whilst you are in the ICU you will undergo the following:

- A check on the status of your breathing and whether you need to remain on the ventilator.
- A check on your vital organs and your vital signs will be measured.
- A note will be made of any changes in the treatment or medication you are receiving.
- A blood sample and, if possible, a urine sample will be taken to check how you are responding to the treatment.
- Your medical chart will be reviewed to see if you have had any new illness or injury.

In addition, on Days 2 to 14 a blood sample will be taken to check on specific effects of the study medication (a sample for central testing of biomarkers). And on Day 7 you will have an ECG.

On your last day in the ICU, you will be given a full physical examination, and a blood sample will be taken to test if you have developed antibodies to the study medication.

If you leave the ICU before Day 28, the study doctor will check the following on Day 28:

- A check on the status of your breathing and whether you need to remain on the ventilator.
- Whether you still need hospital care.
- A full physical examination
- Your vital signs will be measured.
- A note will be made of any treatment you are receiving.
- Your medical chart will be reviewed to see if you have had any new illness or injury.
- A blood sample and, if possible, a urine sample will be taken to check how you are responding to the treatment.

Follow-up

At 3, 6 and 12 months after the start of the study you will have a follow-up to check whether you need to remain on the ventilator and whether you still need hospital or ICU care. Your medical chart will be reviewed to see if you have had any new illness or injury. The 3-month check can be via telephone contact.

At 6 and 12 months you will be asked to complete a short questionnaire on your quality of life, and your lung capacity will be tested by measuring how much air you can breathe out in the first second, after breathing in as much as you can. There will also be a test of how well you can exercise by measuring how far you can walk in 6 minutes. You will also have an ECG.

If during the follow-up period you withdraw your consent and you do not let the study doctor know, then the study doctor may contact others such as your primary doctor or check public records to find out if you are still alive. Attempts may also be made to contact you or your relatives to ascertain this information. If you do not want this information about you to be collected, you may record your objection with your study doctor at any time.

Withdrawal

If you withdraw from the study voluntarily or are withdrawn involuntarily before Day 28, you will be asked to have the tests, examinations and follow-up questions described above for Day 28. You have the right to refuse these tests and examinations. After Day 28, no further follow-up will be done.

Blood Tests and Urinalysis

As part of your treatment your doctor will be taking blood samples from you regularly. In addition to these routine samples, blood will be taken specifically for the purposes of this study to centrally analyse biomarkers to find out how you are responding to the treatment. The amount of blood taken will depend on how long you remain in the ICU. The maximum amount of blood taken for these tests would be about 6-7 tablespoons or around 100 ml over the maximum test period of 28 days.

If you are a woman who may be able to have children, there will also be a urine or blood pregnancy test at screening.

All your test results are confidential and will be disclosed only as required by law. Your blood and urine samples will be destroyed after the laboratory tests are completed.

Your blood sample for biomarker testing will be stored with similar samples at the central laboratories Euro Diagnostica AB - Wieslab AB in Sweden and MediCity Research Laboratory, University of Turku in Finland until end of year 2019. Your sample will not be labelled with your name, but only with a code that is assigned to you when you are enrolled in the study. You have the right to be informed of any plans for new analyses on retained identifiable samples that are not currently foreseen and you have the right to refuse further analyses. You also have the right to withdraw your consent to use/store your samples (including requesting destruction of the sample) as long as the link to your identity is unbroken, and without explaining the reasons of your decision.

What will I have to do?

If you take part in this study you should comply with all study procedures carried out by study medical and nursing staff, over the potential study duration of up to 376 days.

When deciding whether to participate, consider whether you are able and willing:

- To follow the study rules
- To commit the time required to keep appointments
- To tell the study doctor truthfully about your complete medical history
- To report any new problems, illnesses, or changes in medication during the study

What will happen at the end of the study?

Study drug treatment period is 6 consecutive days. Interruptions are not allowed and should an interruption occur, the study drug cannot be initiated again. After the study drug is stopped after 6 days of treatment, your study doctor will decide what medical treatment you should receive, following the best standard of care. Your condition is being followed up until 360 days have been completed after your entry to the trial.

What are the potential risks and discomforts?

Recombinant human interferon beta has been used for many years in subjects with multiple sclerosis. The dose of FP-1201-lyo in this study has been chosen to provide optimal efficacy but to minimize side effects.
As with all research studies, the study medication and study procedures may involve unknown risks. Like all medicines, the study medication can cause side effects, although not everybody gets them. The use of FP-1201-lyo alone or in combination with other drugs may include risks that are not known at this time. The study medication may not help control your ARDS.
If you are or become pregnant, or if your partner becomes pregnant, there may be unknown risks to the baby. So far only 55 subjects with ARDS have been treated with interferon beta-1a, so an estimate of the specific risk in subjects with your condition is difficult to make. In the 37 subjects who have taken part in the previous study with FP-1201, the most commonly reported effect related to the study drug was fever, which is commonly seen in subjects treated with interferon. Other commonly reported side effects were insomnia, a fast heart beat, atrial fibrillation (an irregular heart beat), and low haemoglobin (anaemia). In the Japanese study FP-1201-lyo on 18 subjects, the most commonly report adverse event was also fever. Other commonly reported side effects were liver problems (Hepatic function abnormal), a fast heart beat and chills.

There are several known side effects associated with other licenced interferon beta-1a products in the chronic treatment of multiple sclerosis. However, as the treatment is chronic, the treated condition is different and the administration route is different, these are less relevant or not relevant at all in many parts.

Rarely, subjects with multiple sclerosis treated with interferon beta-1a have had serious allergic (hypersensitivity) reactions with a sudden difficulty breathing, which may appear with swelling of the face, lips, tongue or throat, nettle rash, itching all over the body, and a feeling of weakness or faintness.

Other serious side effects reported for interferon beta-1a in the treatment of multiple sclerosis are:
- Liver problems: jaundice (yellowing of the skin or of the whites of the eyes), widespread itching, loss of appetite accompanied by nausea and vomiting and easy bruising of the skin.
- Hepatic failure
- Suicidal thoughts
- Cardiac disease

The majority of adverse reactions observed with interferon beta-1a in the treatment of multiple sclerosis are usually mild and reversible, and respond well to dose reductions.

<u>Adverse Reactions that apply to this kind of treatment</u>
The administration of interferons has been associated with anorexia, dizziness, anxiety, arrhythmias, vasodilation, palpitation, heavy menstrual bleeding (menorrhagia) and irregular uterine bleeding (metrorrhagia). An increased formation of autoantibodies may occur during the treatment with IFN beta.
The relevance and probability of these adverse events associated with a chronic treatment to occur in the treatment of ARDS with a six-day course of FP-1201-lyo is not known.

Your doctor and nurses will be closely observing you to look for and treat any possible side effects.

The use of FP-1201-lyo alone or in combination with other drugs may include risks that are not known at this time.

<u>X-ray examination or computed tomography (CT) of the chest</u>
As part of this study you will have a chest X-ray or CT scan, but these may be done as part of the routine care for a patient with ARDS. The X-ray or CT scan involves exposure to a low level of radiation. The radiation exposure from one chest X-ray is equivalent to the amount of radiation exposure one experiences from our natural surroundings in 10 days, and from a chest CT scan in 6 months to 2 years, depending on the method used.

<u>Blood collections</u>
Blood will be taken from a tube (catheter) that is already in place in a vein as part of standard care. No discomfort should be experienced. However, should the catheter no longer be in place then the insertion of the venous catheter or individual needle sticks for blood sampling may cause some discomfort. When a sample of your blood is drawn, you may experience some temporary discomfort, bruising, swelling and/or, in rare circumstances, infection at the needle site

<u>ECG</u>
You may experience minimal discomfort from the ECG during the attachment and removal of the ECG electrodes to and from the skin.

What are the advantages and disadvantages of participation in the study?

Taking part in this study may or may not benefit you. You may be randomised into either treatment group (placebo versus FP-1201-lyo). However, the main purpose of the study is to demonstrate that the FP-1201-lyo treatment may provide a significant clinical benefit over the current best available treatments.

Are there any alternative treatments?

There are currently no approved medicines for treating moderate or severe ARDS. If you do not participate in this study, you will only receive the standard care offered by your doctor. The current standard treatment for lung injury is mechanical ventilation (in which breathing is controlled by a machine) together with fluid management. These are combined with treatment for the causes of lung injury whether this is infection-related (sepsis) or from trauma or injury.

Will you be informed if new information becomes available during the study?

Sometimes we get new information about the drug. If this happens, your study doctor will tell you in a timely manner and discuss whether you should continue in the study. You may decide not to carry on or you may decide to continue in the study, in which case your doctor may ask you to sign an updated consent form.

Who can you contact with further questions?

You may ask questions about this consent form or the study at any time (before or during the course of the study). If you have additional questions, or experience a research-related injury, contact the study doctor using the details provided in the table on the first page of this information sheet.

If you have a complaint or question about your rights as a research subject, you may contact the [Ethics Committee (EC) or Institutional Review Board (IRB)] using the details provided in the table on the first page of this information sheet. This is a group of scientific and non-scientific individuals who review research studies with the safety and welfare of research subjects in mind.

A description of this clinical trial will be available on http://www.ClinicalTrials.gov, as required by US Law. This Web site will not include information that can identify you. At most, the Web site will include a summary of the results. You can search this Web site at any time.
This web site only shows data in English, but you can request information from the study staff at any time and have access to data that are publicly available.

Are there any costs if you decide to participate?

You will not receive payment for participating in this study, but the study drugs will be made available to you at no charge and you will not be required to pay for any study procedures. You may be reimbursed for any reasonable travel expenses (bus/train/taxi fares) for the 6- and 12-month visits incurred as a result of taking part in this study on production of a receipt.

Who is funding this research?

Faron Pharmaceuticals Ltd (a pharmaceutical company), will be organizing and funding this study. Faron Pharmaceuticals Ltd will pay your study doctor and/or the study site to cover their costs of conducting this study. If applicable, your study doctor will disclose to you any financial links or other interests that he/she may have to the Sponsor.

Are you insured when you participate in the study?

After you have left the ICU, if you have any unexpected symptoms or injuries, and if emergency medical treatment is required, please report it immediately to:
_________________________________ (**Site to insert contact name and number**).
If you have a physical injury or illness directly related to the study medication or study procedure which was properly performed in accordance with the protocol (referred to as a study-related injury), medical treatment will be provided to you, without charge. The Sponsor, Faron Pharmaceuticals Ltd., has insurance to cover study-related injuries according to the national legislation and local law (**modify the sentence to comply with local law**). Your study doctor will explain how you can obtain a copy of these guidelines.

How will your confidentiality be respected and the privacy of your personal information maintained?

You have the right to control the use and disclosure of your personal information. Basic personal information will be recorded including your name, contact details, gender, height, weight and racial origin (to be used only for clinical purposes), as well as information on your medical history, and clinical data collected about your participation in the study. The following people may also access these records:

- Study monitors and auditors, who may work for Faron Pharmaceuticals Ltd or its authorized representatives, who check that the study is being performed correctly and that the information collected about you is accurate;
- Ethical committee that approved this study and ensures that your rights and well-being are safeguarded;
- National and international regulatory authorities involved in keeping research safe for patients;
- Clinical trial recruitment company if you were referred to the study by such a company, for analytical purposes and so they may be compensated.

All personnel accessing your records are required to respect your confidentiality at all times.

To ensure privacy, your name and other identifying information will not be attached to records or samples released for research purposes. Instead, you will only be identified by a code. Only the study doctor and authorized personnel will be able to connect this code to your name, by a list that will be kept securely by the study site for 15 years. Your date of birth and initials may also be recorded to help identify your study record. Your coded data will be forwarded to Faron Pharmaceuticals Ltd and its service providers for activities related to the study e.g. laboratory analysis. It will be transferred into a computer database and processed to allow the results of this study to be analyzed and reported or published. If the results of the study are published, your identity will remain confidential. A list of companies to whom your coded information is transferred is available from Faron Pharmaceuticals Ltd via your study doctor.

Under data protection law [identification of national law] your study site and Faron Pharmaceuticals Ltd shall be jointly responsible as 'controllers' for ensuring that your information is safeguarded. You have the right to access, through your study doctor, all the information collected about you and, if applicable, ask for corrections. But, in order to protect the scientific integrity of the study, the treatment you received in this study needs to remain unknown (= blinded) until the study data is analyzed.

You also have the right to complain about how your information is handled to a supervisory authority that is responsible for enforcing data protection law. A list of European Union supervisory authorities is available here: http://ec.europa.eu/justice/data-protection/article-29/structure/data-protection-authorities/index_en.htm.

Recipients of your information may be in countries that do not provide the same standard of legal protection for your information as in the EU, raising the risk that you will not be able to enforce the above rights and recipient organisations may not be legally required to fully secure your data. Certain international recipients of your information may have signed special contracts to provide legal protection for your transferred information (e.g. so called “Standard Data Protection Clauses”). In any event, all parties involved in the study are required to maintain your confidentiality.

If you should withdraw from the study, data collected prior to your withdrawal may still be processed along with other data collected as part of the study. Normally no new information will be collected for the study database unless you specifically consent to that. However, the law does require that any side-effects you may suffer are documented. To complete the study findings, your long term health status may also be ascertained from publicly available records (unless you object to your study doctor). You have the right to require that any previously retained samples are destroyed.

This study may only be performed by collecting and using personal information on study participants as described in this form, therefore you may only participate in the study if you agree to the collection and use of your information as described here.

If you have any questions, comments or complaints about how your information is handled in this study, or wish to obtain a copy of the Standard Data Protection Clauses, you should firstly contact your study doctor who will be able to direct your query where appropriate to staff responsible for data protection at the Faron Pharmaceuticals Ltd or site, including the site Data Protection Officer.

Has the study received medical or ethical approval?

The Ethics Committee has given this study a positive opinion.

Name of ethics committee:
Telephone number:

Statement of Consent

This clinical study may only be performed by collecting and using your medical information. Data protection laws give you the right to control the use of your personal information. Therefore, by signing this form you specifically authorize your information to be checked, transferred and processed as follows:

- I agree the authorized representatives of Faron Pharmaceuticals Ltd the Ethics Committee and regulatory authorities’ inspectors may review my medical information by direct access to my medical records.
- I agree study data, including my coded medical information, may be used and shared for legitimate study and scientific purposes, including if I do not object, for future use in medical or pharmaceutical research.
- I agree study data may be transferred to other countries for processing, including countries not covered by the data protection legislation.
- I have received verbal information on the above study and have read and understand the attached written information.

- I have been given the chance to discuss the study and ask questions and I am satisfied with the explanations provided
- I voluntarily agree and consent to participate in this study, including all assessments and taking of blood samples (except for genetic testing).
- I understand that I am free to withdraw at any time. I understand that if I choose to not participate or to withdraw, my current medical care will not be affected by this decision.
- I agree that my primary doctor may be informed of my participation in this study.
- I agree that my primary doctor may be asked to provide information about my medical history.
- I agree that my personal data, including data relating to my physical or mental health or condition, and ethnic origin, may be used as described in this consent form.
- I understand that I and/or my legal representative will receive and may keep a copy of this signed and dated consent form.
- By signing and dating this consent form, I have not waived any of the legal rights that I would have if I was not a patient in a medical research study.

______________________	__________	______	AM/PM	____________________
Signature of Subject	Date (mm/dd/yyyy)	Time		Printed Name of Subject
______________________	__________	______	AM/PM	____________________
Signature of Witness*	Date (mm/dd/yyyy)	Time		Printed Name of Witness
______________________	__________	______	AM/PM	____________________
Signature of Legal Representative	Date (mm/dd/yyyy)	Time		Printed Name of Legal Representative

____

Relationship of Personal Legal Representative to Subject (e.g., father/mother/son etc.)

____

Job Title of Professional Legal Representative

* An impartial witness should sign here if the subject speaks and understands [English], but does not read and write. Note that this refers to the subject's literacy, and not being unable to read or write because the subject is unconscious.

1. STATEMENT OF PERSON CONDUCTING INFORMED CONSENT DISCUSSION

I, the undersigned, certify that to the best of my knowledge, the subject signing this consent form had the study fully and carefully explained and clearly understands the nature, risks, and benefits of participation in this research study.

______________________	____________	________ AM/PM	______________________
Signature of Investigator (or other Person Obtaining Consent)	Date (mm/dd/yyyy)	Time	Printed Name of Person Obtaining Consent

CONSENT OF THE PATIENT TO CONTINUE TO BE IN THE STUDY

Your legal representative gave his/her consent for you to be in this research study. This is because you were not able to make your own decision due to your illness. Your condition has now improved. You are being asked to decide whether to continue to be in this study. Your decision is voluntary.

You have read the information in this form and someone has explained to you which study procedures will be continuing. All of your data collected so far will be included in the analysis of the study. Your questions have been answered to your satisfaction. You believe you understand all of the information about this study. You have decided to continue taking part in this study.

______________________	____________	______________________
Signature of Subject	Date (mm/dd/yyyy)	Printed Name of Subject

______________________	____________	______________________
Signature of Person Obtaining Consent	Date (mm/dd/yyyy)	Printed Name of Person Obtaining Consent

If you decide **not** to give your consent to continue to be in the study, you must also decide what happens to the data that have been collected so far. You have three options. Please check one of the corresponding boxes below:

You have decided you do **not** want to continue taking part in this study.

□	I choose to keep my study data that has been collected so far in the analysis
□	I do not want any of my study data to be included in the analysis except data gives information on how safe the drug is.
□	I do not want any of my study data to be included in the analysis

______________________	____________	______________________
Signature of Subject	Date (mm/dd/yyyy)	Printed Name of Subject

______________________	____________	______________________
Signature of Investigator (or other Person Obtaining Consent)	Date (mm/dd/yyyy)	Printed Name of Person Obtaining Consent
